# Incidence and risk factors of postoperative complications in patients with tuberculosis-destroyed lung

**DOI:** 10.1186/s12890-021-01641-0

**Published:** 2021-08-21

**Authors:** Hongyun Ruan, Fangchao Liu, Ming Han, Changfan Gong

**Affiliations:** 1 Department of Cardiopulmonary Function, Beijing Chest Hospital, Capital Medical University, No 9, Beiguan Street, Tongzhou District, Beijing, 101149 People’s Republic of China; 2 Department of Academic Research, Beijing Chest Hospital, Capital Medical University, No 9, Beiguan Street, Tongzhou District, Beijing, 101149 People’s Republic of China; 3 Department of Thoracic Surgery, Beijing Chest Hospital, Capital Medical University, No 9, Beiguan Street, Tongzhou District, Beijing, 101149 People’s Republic of China

**Keywords:** Tuberculosis, Destroyed lung, Postoperative complication, Risk factor

## Abstract

**Background:**

The purpose of this study was to determine risk factors of postoperative complications in tuberculosis-destroyed lung (TDL) patients.

**Methods:**

We retrospectively analyzed the data from all consecutive TDL patients undergoing surgical treatment at the Beijing Chest Hospital from January 2001 to September 2019.

**Results:**

Of 113 TDL cases experiencing surgery, 33 (29.2%) experienced postoperative complications. The patients with low BMI were more likely to have postoperative complications compared to those with normal BMI, whereas a significant lower rate of postoperative complications was noted in patients with BMI ≥ 25 kg/m^2^. In addition, significant increased risk was observed in patients with smoking history. We found that the patients with underlying infection, including aspergillus and nontuberculous mycobacteria (NTM), had significantly higher odds of having postoperative complications compared with those without underlying infection. The anaemia was another important independent factor associated with postoperative complication. Patients with blood transfusion above 1000 mL had a strongly increased frequency of postoperative complications than patients with blood transfusion below 1000 mL.

**Conclusion:**

In conclusion, our data demonstrate that approximate one third of TDL patients experience postoperative complications in our cohort. Patients with low BMI, anaemia, tobacco smoking, and coinfected aspergillus or NTM are at markedly higher risk to experience postoperative complications after pneumonectomy.

## Introduction

Tuberculosis (TB) remains a significant cause of global morbidity and mortality [1, 2].In 2019, 10.0 million people developed TB and 1.4 million people died from the disease, with most cases in developing countries [1]. China has approximately 0.83 million new cases of TB every year, more than any country except India and Indonesia [1]. Despite great achievement in TB control over past decades, detection of laboratory-confirmed cases had stagnated at around 30% of the notified cases [3]. As a consequence, delay in diagnosis of pulmonary TB leads to increasing severity, mortality and transmission in the community [4].

Pulmonary TB can cause variable changes in pulmonary parenchyma, ranging from pulmonary infiltrate to parenchymal lung devastating [5]. Tuberculosis-destroy lung (TDL) is one of the most devasting sequelae of TB [6]. Decreased lung and airway volume in patients with TDL are associated with progressive lung function decline, respiratory failure and pulmonary disabilities [6]. Despite resulting in fatal outcomes, there is no formal treatment guidelines for TDL patients. Currently, pneumonectomy for destroyed lung has always been an alternative for the treatment of TDL patients, especially those combined with massive hemoptysis and repetitive infection [7]. The few studies have demonstrated that the high mortality of patients undergoing surgery is majorly due to concurrent medical problems [7]). Identifying which patients are at greatest risk of developing complications and which types of complications are life threatening is essential for reducing the mortality of TDL. Unfortunately, little is known about this aspect in affected patients, hampering the effective interventions for individuals at high risk.

To address this concern, we conducted an observational retrospective cohort study among TDL patients undergoing surgery over the last18 years. Our objectives were to determine risk factors of postoperative complications in TDL patients.

## Material and methods

### Study design

This study is a single-centre retrospective analysis of postoperative complications in TDL patients. In this study, TDL was defined as pulmonary destruction caused by the sequelae of tuberculosis in the past, and X-rays or chest CT show the coexistence of fibrotic cavities and a caseous focus or are accompanied by segmental atelectasis or bronchiectasis [8, 9]. We obtained data from all consecutive TDL patients undergoing surgical treatment at the Beijing Chest Hospital, a specialized hospital for tuberculosis and thoracic tumor from January 2001 to September 2019. Exclusion criteria included: (1) coexistence of thoracic tumor; (2) thoracic deformity; (3) chronic heart failure.

In our study, the proportion of preoperative HIV screening population was 56.6% (64/113), and all of them were negative. In the early years, there was no routine screening for HIV infection before surgery. Demographic and clinical data were extracted from the database of the medical record, including age, sex, biochemical investigation results preoperative comorbidities, operative treatment, drug administration and postoperative complications. The study design complies with the Helsinki statement on research ethics and was approved by the Ethics Committee of Beijing Chest Hospital, Capital Medical University. The written consent was waived due to full anonymization of data.

### Clinical parameters

We assessed the clinical parameters at baseline as follows: smoking history, previous medical history, blood examination, pulmonary function test, and chest computed tomography. Body Mass Index (BMI) was used to classify individuals as underweight (BMI < 18.5), normal weight (18.5 ≤ BMI < 25.0), and overweight (BMI ≥ 25.0) [10]. The smoking index was defined as the number of cigarettes per day multiplied by the number of smoking years [11]. The heavy smoker was referred to that with a smoking index higher than 400. Multidrug-resistant TB (MDR-TB) was defined as TB caused by a *Mycobacterium tuberculosis* strain that was resistant to rifampin and isoniazid; extensively drug-resistant TB (XDR-TB) was defined as MDR-TB with additional resistance to both any fluroquinolone and any second-line injectable drug [3]. Pulmonary function test reference values were calculated according to the method by European respiratory society/American thoracic society [12].

The surgical indication of TDL was that the lesions were confined to the ipsilateral lung with the following conditions were met: TDL were located in the ipsilateral lung; massive hemoptysis; persistent positive of sputum smear and/or culture of Mycobacterium tuberculosis; antituberculosis treatment was ineffective or intolerable; recurrent infection of TDL. Surgical contraindications were as follows: respiratory failure in preoperative blood gas analysis; serious comorbidities; bilateral involvement of lesions. Whether or not to take surgical treatment for TDL patients was discussed and decided by a team of experts from radiology, thoracic surgery and anesthesiology [13]. The time span from chest CT diagnosis to operation was 0–120 months (median 2 months).

Postoperative complications included death during same admission or within 30 days after surgery when discharged, heart failure, respiratory failure with mechanical ventilation, sepsis, and postoperative bleeding.

### Statistical analysis

The continuous variables were expressed as mean (SD) or median (range), and categorical variables as count (%). We assessed the values of laboratory results employing student t test or Wilcoxon rank-sum test depending on whether underlying distribution of variable was normal. The chi-square test was conducted to estimate risk factors for non-responder. We used SPPS version 20.0 (IBM, Armonk, NY) to conduct all calculations. The difference was declared significant, if two-sided P values less than 0.05.

## Results

### Patients

**Figure1:**
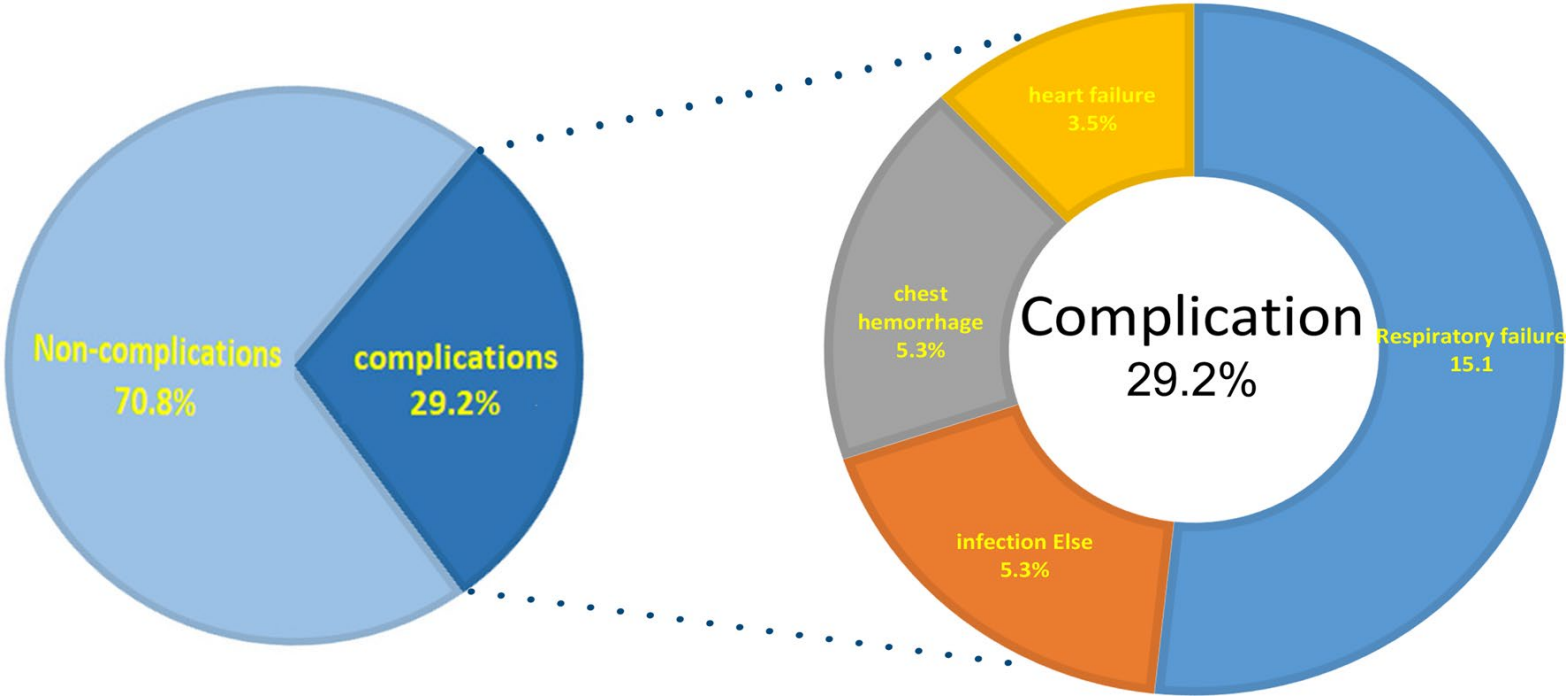
The distribution of postoperative complications in patients with tuberculosis-destroyed lung. The pie chart (left) showed the proportion of complications and non-complications in TDL patients. The pie chart (right) showed the proportion of different complications, include secondary respiratory failure, other site infection, chest bleeding and heart failure

A total of 113 TDL cases experiencing surgery were enrolled from January 2001 to September 2019 in our analysis. The mean age of these cases was 39.2 years, and 45.1% were male. Overall, 33 (29.2%) experienced postoperative complications. The most frequently observed complication was respiratory failure (n = 17, 15.1%), followed by 6 (5.3%) infection else, 6 (5.3%) Chest hemorrhage and 4 (3.5%) heart failure (Fig. 1). 23% (26/113) of the patients did not receive preoperative pulmonary rehabilitation guidance because of emergency operation, and all other patients conducted preoperative pulmonary rehabilitation.

### Demographic and clinical characteristics associated with postoperative complications in TDL patients

The comparison of demographic and clinical characteristics of TDL patients stratified to the presence of postoperative complications is summarized in Table 1. The patients with low BMI were more likely to have postoperative complications compared to those with normal BMI [adjusted odds ratio (aOR): 6.333, 1.957–20.500], whereas a significant lower rate of postoperative complications was noted in patients with BMI ≥ 25 kg/m^2^ (aOR: 0.726, 0.120–4.381). Although heaviness of smoking index had no impact on the presence of postoperative complications, significant increased risk was observed in patients with smoking history (aOR: 5.592, 1.519–21.334). In addition, we found that the patients with underlying infection, including aspergillus and nontuberculous mycobacteria (NTM), had significantly higher odds of having postoperative complications compared with those without underlying infection (aOR: 4.036, 1.385–11.766).

**Table 1:** Analysis of demographic and clinical characteristics of TDL patients stratified to the presence of postoperative complications

Variables	Total (*n* = 113%)	Non-complication group (*n* = 80%)	Complication group (*n* = 33%)	Crude OR (95% CI)	Adjusted OR (95% CI)
*Sex*					
Male	51 (45.1)	31 (60.8)	20 (39.2)	2.432 (1.060–5.580)	
Female	62 (54.9)	49 (79.0)	13 (21.0)	Ref	
*Age group (years)*					
≤ 65	110 (97.3)	79 (71.8)	31 (28.2)	Ref	
> 66	3 (2.7)	1 (33.3)	2 (66.7)	5.097 (0.446–58.250)	
*BMI (kg/m*^*2*^*)*					
< 18.5	24 (21.2)	10 (41.7)	14 (58.3)	4.859 (1.834–12.875)	6.333 (1.957–20.500)
18.5–24.9	76 (67.3)	59 (77.6)	17 (22.4)	Ref	Ref
≥ 25	13 (11.5)	11 (84.6)	2 (15.4)	0.631 (0.127–3.126)	0.726 (0.120–4.381)
*Smoking history*					
No	97 (85.8)	73 (75.3)	24 (24.7)	Ref	
Yes	16 (14.2)	7 (43.8)	9 (56.2)	3.911 (1.315–11.633)	5.592 (1.519–21.334)
*Smoking index*					
< 400	4 (25.0)	2 (50.0)	2 (50.0)	Ref	
≥ 400	12 (75.0)	5 (41.7)	7 (58.3)	1.4 (0.144–13.568)	
*Alcohol abuse*					
No	108 (95.6)	77 (71.3)	31 (28.7)	Ref	
Yes	5 (4.4)	3 (60.0)	2 (40.0)	1.656 (0.264–10.397)	
*Comorbidities*					
No	91 (80.5)	67 (83.8)	24 (70.0)	Ref	
Yes	22 (19.5)	13 (14.1)	9 (30.0)	1.933 (0.733–5.095)	
*Course of disease*					
< 12 months	17 (15.0)	13 (76.5)	4 (23.5)	Ref	
12–120 months	66 (58.4)	46 (70.0)	20 (30.0)	1.413 (0.410–4.871)	
> 120 months	30 (26.5)	21 (70.0)	9 (30.0)	1.393 (0.355–5.459)	
*Coinfection*					
No	80 (70.8)	64 (80.0)	16 (20.0)	Ref	Ref
Aspergillus or NTM	33 (29.2)	16 (46.7)	17 (53.3)	4.250 (1.771–10.198)	4.036 (1.385–11.766)
*MDR*					
No	100 (88.5)	71 (71.0)	29 (29.0)	Ref	
Yes	13 (11.5)	9 (69.2)	4 (30.8)	1.088 (0.310–3.815)	
*Severity of TDL*					
Lobe = 1	31 (27.4)	22	9	Ref	
Lobe > 1	82 (72.6)	58	24	1.011 (0.407–2.512)	
Lung function*					
FVC%pred		65.6 ± 16.7	61.5 ± 19.2	0.986(0.957–1.015)	
FEV_1_%pred		57.5 ± 18.3	55.9 ± 18.0	0.995(0.969–1.022)	
MVV%pred		56.8 ± 19.8	55.6 ± 17.1	0.997(0.972–1.023)	
DLCO%pred		59.5 ± 16.5	55.8 ± 8.7	1.018(0.995–1.042)	

Data are presented as mean (SD) or n (%) or median (range)

Comorbidities include hypertension, diabetes and coronary heart disease. FEV_1_% pre, forced expiratory volume in one second as a percentage of predicted value; FVC % pre, forced vital capacity as a percentage of predicted value; MVV % pre, maximal voluntary ventilation as a percentage of predicted value; DLCO, lung diffusion capacity of predicted. In view of small sample size of patients with comorbidities, they are combined into one group for statistical analysis; * 23% (26/113) of the patients with massive hemoptysis underwent emergency surgery without preoperative pulmonary function examination, and 87 cases had pulmonary function examination results

*TDL* tuberculosis destroyed lung, *BMI* body mass index, *OR* odds ratio, *CI* confidence interval, *NTM* nontuberculous mycobacteria, *MDR* multidrug resistance

In addition, patients with decreased C-reaction protein (CRP) were more likely to have postoperative complication (OR: 3.360, 1.171–9.00). Complications occurred more frequently in patients with low albumin level than those with normal albumin level (OR: 3.088, 1.294–7.369). The anaemia was another important factor associated with postoperative complication (OR: 10.50, 2.051–53.748), which was also a significant predictor of postoperative complication after adjustment (aOR: 9.634, 1.270–73.066) (Table 2).

**Table 2:** Analysis of laboratory and clinical examinations of TDL patients stratified to the presence of postoperative complications

Variables	Total (*n* = 113%)	Non-complication group (*n* = 80%)	Complication group (*n* = 33%)	Crude OR (95% CI)	Adjusted OR (95% CI)
*Electrocardiogram*					
Normal	67 (59.3)	46 (68.7)	21 (31.3)	Ref	
Abnormal	46 (40.7)	34 (73.9)	12 (26.1)	0.773 (0.335–1.784)	
*Leukocyte (*× *10*^*9*^*/L)*					
< 0.4	8 (7.1)	6 (75.0)	2 (25.0)	0.853 (0.161–4.514)	
0.4–1.0	89 (78.8)	64 (71.9)	25 (28.1)	Ref	
> 1.0	16 (14.2	10 (0.625)	6 (37.5)	1.536 (0.505–4.673)	
*Creatinine (μmol/L)*					
45–104	11 (9.7)	6 (54.5)	5 (45.5)	2.202 (0.622–7.795)	
< 45	102 (90.3)	74 (72.5)	28 (27.5)	Ref	
*CRP (mg/L)*					
0–5	35 (31.0)	30 (85.7)	5 (14.3)	Ref	
> 5	78 (69.0)	50 (64.1)	28 (35.9)	3.360 (1.171–9.000)	
*Platelet (*× *10*^*9*^*/L)*					
Normal	81 (71.7)	59 (72.8)	22 (27.2)	Ref	
Decreased	32 (28.3)	21 (65.6)	11 (34.4)	1.405 (0.584–3.382)	
*Albumin (g/L)*					
≥ 35	32 (28.3)	17 (53.1)	15 (46.9)	3.088 (1.294–7.369)	
< 35	81 (71.7)	63 (77.8)	18 (22.2)	Ref	
*Hemoglobin (g/L)*					
> 9	9 (8.0)	2 (22.2)	7 (77.8)	10.50 (2.051–53.748)	9.634 (1.270–73.066)
≤ 9	104 (92.0)	78 (75.0)	26 (25.0)	Ref	
*Blood glucose (mmol/L)*					
3.9–6.1	98 (86.7)	72 (90.0)	26 (10.0)	Ref	
> 6.1	15 (13.3)	8 (53.3)	7 (46.7)	2.423 (0.799–7.346)	
*pH*					
7.35–7.45	78 (94.0)	54 (69.2)	24 (30.7)	Ref	
> 7.45	5 (6.0)	2 (40.0)	3 (60.0)	1.837 (0.728–4.639)	
*PaO*_*2*_* (mmHg)*					
< 80	13 (15.7)	9 (69.2)	4 (30.8)	0.638 (0.185–2.201)	
≥ 80	70 (84.3)	47 (67.1)	23 (32.9)	Ref	
*PaCO*_*2*_* (mmHg)*					
≤ 45	56 (67.5)	40 (75.0)	16 (28.6)	Ref	
> 45	27 (32.5)	16 (59.3)	11 (40.7)	1.719 (0.657–4.498)	

Data are presented as mean (SD) or n (%) or median (range)

*TDL* tuberculosis destroyed lung, *CRP* C-reactive protein, *OR* odds ratio, *CI* confidence interval

We also analysed the intraoperative indicators stratified to the presence of complications or not. As summarized in Table 3, patients with blood transfusion above 1000 mL had a strongly increased frequency of postoperative complications than patients with blood transfusion below 1000 mL (OR: 5.277, 2.052–13.316), which was also a significant predictor of postoperative complication after adjustment (aOR: 4.911, 1.647–14.639). In contrast, the operation time, volume of intraoperative bleeding and operative mode had no effect on the presence of postoperative complications.

**Table 3:** Analysis of surgical operations of TDL patients stratified to the presence of postoperative complications

Variables	Total (*n* = 113%)	Non-complication group (*n* = 80%)	Complication group (*n* = 33%)	Crude OR (95% CI)	Adjusted OR (95% CI)
*Operation time (hour)*					
≤ 4	65 (57.5)	49 (75.4)	16 (24.6)	Ref	
> 4	48 (42.5)	31 (64.6)	17 (35.4)	1.679 (0.741–3.804)	
*Volume of intraoperative bleeding (mL)*					
≤ 1000	75 (66.4)	57 (76.0)	18 (24.0)	Ref	
> 1000	38 (33.6)	23 (60.5)	15 (39.5)	2.065 (0.892–4.779)	
*Volume of blood transfusion (mL)*					
≤ 1000	87 (77.0)	69 (79.3)	18 (20.7)	Ref	
> 1000	26 (23.0)	11 (42.3)	15 (57.7)	5.277 (2.052–13.316)	
*Operative mode*					
Lobectomy	35 (31.0)	20 (57.1)	15 (42.9)	Ref	
Pneumonectomy	78 (69.0)	60 (76.9)	18 (23.1)	0.400 (0.171–0.938)	

Data are presented as mean (SD) or n (%) or median (range)

*TDL* tuberculosis destroyed lung

## Discussion

Clinical management of TDL patients remains challenging in view of the lack of formal treatment guidelines [6, 14]. Pneumonectomy for destroyed lung is of fundamental importance to improve clinical outcomes for TDL patients; whereas the presence of postoperative complications increases the burden of resource use, and affect late survival [7]. This report described our analysis for risk factors associated with postoperative complications among TDL patients.

Our data demonstrate that approximate one third of TDL patients experience postoperative complications in our cohort. A detailed analysis of case series in Shanghai revealed that the proportion of destroyed lung patients experiencing postoperative complication was 18% [7], a significantly lower than our observation. One possible explanation for this difference is that the etiology of destroyed lung is not only tuberculosis, but also multiple pulmonary diseases, such as bronchiectasis and bronchocele [7]. Considering that chronic TB infection leaded to poor physical condition, the TDL patients are more likely to have postoperative complication than others with destroyed lung due to other etiologies. In another study, the incidence of postoperative complications in patients with TDL was 18.6% [8], which was also lower than that in our study (29.2%), which may be due to the older age of patients, the higher proportion of pulmonary aspergillosis and the higher proportion of pneumonectomy in our study. Therefore, intensive care is required for TDL patients after pneumonectomy to reduce the incidence of complications.

It is well recognized that low BMI and malnutrition were independent risk factors for postoperative adverse outcomes [15, 16]. Similar findings were observed in our analysis that low BMI and anaemia had the greatest risk of postoperative complications. Of note, all these indicators reflect the poor nutritional status of TDL patients. On the one hand, experimental studies revealed that malnutrition had a negative impact on the host immune system [17], thus delaying the activation of adequate response to infection. As a consequence, these patients are at increased risk for postoperative infections. On the other hand, a previous report by Pakasi and colleagues showed that BMI was an indicator for clinical severity of pulmonary TB considering that this disease is a wasting disease [18]; namely, the patients with low BMI were prone to have more severe TB, which is responsible for the presence of postoperative complications. Our findings highlight that preoperative nutritional treatment would provide benefit to reduce postoperative complications for TDL patients.

Consistent with other studies, smokers had an increased risk of postoperative complications in our cohort. One of the possible mechanisms to explain this relationship is that tobacco smoking causes chronic changes in pulmonary, cardiovascular and immune function. In addition, we found a strong association between underlying aspergillus and NTM infections and postoperative complications. Despite typical radiologic findings of pulmonary TB and aspergillus or NTM [13, 19], it is difficult to diagnose these diseases when they coexist in the same patient. Our finding indicated that the misdiagnosis of these concurrent infections resulted in elevated risk for developing serious complications after pneumonectomy. In view of this point, we emphasize the importance of precise diagnosis of infections that occurred concurrently with pulmonary TB.

This study has several limitations. First, despite enrollment of patients meeting inclusion criteria over two decades, the small sample size may weaken the reliability of our conclusions. Further study is urgently required to confirm the risk factors associated with postoperative complications for TDL patients in our study. Second, in the context of the effect of nutritional status on adverse outcomes, it could be more useful to determine the specific mechanisms by which individual nutrients affect the immune system. Because we only obtained conventional laboratory data, we could not elucidate the relationship between each nutrient and presence of postoperative complications. Third, due to the lack of follow-up data, we are not able to evaluate whether the postoperative complications have a significant effect on long-term survival.

In conclusion, our data demonstrate that approximate one third of TDL patients experience postoperative complications in our cohort. Patients with low BMI, anaemia, tobacco smoking, and coinfected aspergillus or NTM are at markedly higher risk to experience postoperative complications after pneumonectomy. Our findings emphasize the importance of early interventions in risk groups to prevent adverse outcomes in TDL patients.

## Data Availability

The datasets used and/or analysed during the current study available from the corresponding author on reasonable request.
